# Differential Retrospective Analysis in Oral Cancerous, Pre-cancerous, and Benign Tissue Biopsies

**DOI:** 10.7759/cureus.24956

**Published:** 2022-05-13

**Authors:** Lampros Goutzanis

**Affiliations:** 1 Department of Oral and Maxillofacial Surgery, National and Kapodistrian University of Athens, Dental School, Athens, GRC

**Keywords:** prevalence, oral cancer, pre-cancerous lesions, cysts, oral lesion, oral biopsy

## Abstract

Background/Aim

Oral epithelia demonstrate a broad spectrum of pre-cancerous, cancerous, and benign lesions. The aim of this study was to record and analyze the prevalence of various oral and intraosseous lesions, highlighting malignancies that are hard to clinically identify as such too.

Materials and methods

A series of 536 oral lesions were collected covering a period of 8.5 years. Epidemiological and clinico-histopathological data were stratified and analyzed retrospectively.

Results

According to extensive differential analysis, the male to female ratio for oral squamous cell carcinoma was estimated at 1:1, for pre-cancerous lesions at 1:2, and for lichen planus at 1:5. The prevalent diagnostic category were cysts (n = 223, 41.6%). The biological behavior of lesions differed among anatomic sites (P<0.001). Concordance between clinical suspicion of pre-cancerous or malignant lesions and histological verification was 96.4% (P<0.001).

Conclusions

Primary intraosseous squamous cell carcinoma, acinic cell carcinoma, clear cell myoepithelial carcinoma, aggressive osteoblastoma/parosteal osteosarcoma, and undifferentiated carcinoma raised no clinical suspicion of malignancy reflecting the importance of training in oral biopsy taking.

## Introduction

Tissue biopsy determines the final diagnosis of a lesion based on specific histological features. In fact, tissue biopsy confirms or excludes clinical suspicion of malignancy, it provides grading and staging classification and plays a pivotal role in further diagnostic and therapeutic strategies. Furthermore, biopsy findings are of irrefutable legal medical value [1-2]. The American Academy of Oral and Maxillofacial Pathology (AAOMP) considers biopsy as the gold standard diagnostic procedure, which is within the scope of practice of general dentists [3].

Oral lesions encompass a wide range of various pathologies including reactive, infectious, immunologic, developmental conditions, benign tumors and tumor-like lesions, potentially malignant disorders, and various types of malignancies. The frequency distribution of these lesions between men and women is different in every decade of life [4]. Their prevalence has been documented by studies that are mostly limited to certain groups of patients such as pediatric populations [5-6], young and middle-aged adults [2], and specific conditions such as mucoceles, intra-osseous lesions, odontogenic cysts, or tumors [7-11]. A relatively small number of studies from Europe and the USA focus on the spectrum and frequency of histologically confirmed diagnosis of oral and/or maxillofacial biopsies in a wide range of age groups during the last decades [4,12-14].

Due to the extended number and large variety of clinical and radiographic appearance of oral and jaw pathologies these lesions may be misleading for clinicians in discerning their nature (benign or malignant) and in reaching the correct clinical diagnosis. The objective of the current study was (i) to record the baseline prevalence and the distribution of oral mucosal and intra-osseous lesions in a Greek civil population comprising various age groups, (ii) to document the prevalence of these lesions according to patient gender and age, and (iii) to focus on cancerous lesions that are hard to clinically identify as such.

## Materials and methods

Study design and sample collection

This is a cross-sectional study based on a survey of the biopsy records and conclusive pathology reports of oral and peri-oral lesions resulting from biopsies performed by a single surgeon in an oral and maxillofacial surgery unit during a period of 8.5 years (1/1/2012 - 31/7/2020). Most biopsies were referrals from general dentists and the rest were referrals from oral pathologists and endodontists. The vast majority of histo-pathological diagnoses were conducted by the Department of Oral Pathology and Medicine (School of Dentistry) and the First Department of Pathology (School of Medicine) of the National and Kapodistrian University of Athens, Greece (NKUA). A few histopathological diagnoses were conducted by Istomedica (a private pathology laboratory, Athens, Greece). A total number of 536 oral and maxillofacial lesions were collected from 545 biopsies corresponding to 497 patients.

Tissue biopsy management

The corresponding archival, paraffin-embedded tissue blocks were fixed in 10% neutral-buffered formalin. After microtome-based sectioning, hematoxylin and eosin (H&E)-stained slides were reviewed for determination of the histopathological type and grade classification, according to the histological typing criteria of 2017 World Health Organization (WHO) guidelines [15]. Concerning the differential diagnosis process, immunohistochemistry (IHC) assays were implemented, including predominantly cytokeratins and cell proliferation markers such as ki-67.

Classification of tumors

Descriptive Statistics

The lesions were identified according to the 2017 WHO classification of odontogenic and maxillofacial bone tumors [15] and the 2017 WHO histological classification of tumors of the oral cavity and mobile tongue [15]. Lesions were also classified into eight diagnostic categories according to the definite histological diagnosis. Further classification was also made based on their biological behavior as follows: a. malignant lesions, comprising malignant tumors and lymphomas, b. pre-cancerous lesions, comprising the histological diagnoses of epithelial dysplasia and epithelial and pseudoepitheliomatous hyperplasia, with a clinical diagnosis of oral leukoplakia (OL), c. benign lesions with a tendency to recur, comprising the histological diagnoses of Langerhans cell histiocytosis (LCH), ameloblastoma and odontogenic keratocyst (OKC), and d. benign lesions (all other histological diagnoses). The distribution of the population was defined by gender, age, and anatomic site of the lesion. Mean values and standard deviation (SD) for age were also calculated.

Statistical analysis

All results were subjected to statistical analysis in terms of the two-tailed Fisher’s exact test for categorical data and the Mann-Whitney U test for quantitative data. Cohen’s kappa coefficient (κ) was also determined, as well as sensitivity, specificity, and positive and negative prognostic values of the clinical identification of lesions into benign and pre-cancerous or malignant versus histopathological definite diagnosis. Differences between groups were assessed by using SPSS v21.0 (IBM Corp, Armonk, NY) with P<0.05 as the threshold of statistical significance.

## Results

Out of 545 biopsies, 74 (13.6%) were incisional biopsies and 471 (86.4%) were excisional biopsies. For seven out of 536 lesions (1.3%), no age could be determined. The mean age (± standard deviation, SD) of patients was 50.5 (±17.9) years (range: 9 - 90 years). Considering the decade of life, lesions were distributed as follows: one (0.2%) lesion was present in the 0-9-year age group, 31 (6.2%) in the 10-19-year age group, 41 (8.2%) in the 20-29-year age group, 74 (14.9%) in the 30-39-year age group, 92 (18.5%) in the 40-49-year age group, 112 (22.5%) in the 50-59-year age group, 98 (19.7%) in the 60-69-year age group, 57 (11.5%) in the 70-79-year age group, 22 (4.4%) in the 80-89-year age group, and one (0.2%) in the 90-99-year age group.

Considering gender, 260 lesions (48.5%) were collected from male (M) and 276 (51.5%) from female (F) patients (M:F = 0.94:1). Mean age was statistically significantly different between the two genders, namely, 46.9 (±17.4) years for men ranging from 9-90 years and 54.0 (±17.7) years for women ranging from 11-88 years (P<0.001).

The vast majority of lesions (n = 223, 41.6%) were categorized as cysts of the jaws and oral soft tissues (jaw:oral soft tissue cysts ratio was 6.69:1), followed by benign tumors and tumor-like lesions (n = 144, 26.9%) and inflammatory diseases (n = 52, 9.7%). Potentially malignant lesions (PMLs) and pre-cancerous lesions accounted for 5.0% of all lesions (M:F = 1:2) (Table 1).

**Table 1:** Number of diagnoses by diagnostic category from January 2012 to July 2020 ^a ^Comprising the following diagnoses: lingual tonsil, minor salivary gland tissue, epithelial hyperplasia (with no clinical oral leukoplakia), scar tissue, cortical bone, normal tissue, hydropic degeneration of basal cells, and subcutaneous tissue

			All lesions			Male (♂)			Female (♀)			M:F ratio		Mean age (± SD)	
Main diagnostic categories			n	(%)		n	(%)		n	(%)					
I.	Benign tumors and tumor-like lesions		144	(26.9%)		52	(20.0%)		92	(33.3%)		0.57		51.0	(± 19.0)
II.	Malignant tumors		28	(5.2%)		14	(5.4%)		14	(5.1%)		1.00		67.9	(± 11.9)
III.	Potentially malignant lesions (PMLs) – Pre-cancerous lesions		27	(5.0%)		9	(3.5%)		18	(6.5%)		0.50		55.1	(± 12.8)
IV.	Cysts of the jaw and oral soft tissues		223	(41.6%)		147	(56.5%)		76	(27.5%)		1.93		44.0	(± 16.0)
V.	Inflammatory diseases		52	(9.7%)		24	(9.2%)		28	(10.1%)		0.86		52.7	(± 15.4)
VI.	Pathology induced by drugs and other factors		27	(5.0%)		5	(1.9%)		22	(8.0%)		0.23		66.2	(± 13.9)
VII.	Oral manifestations of systemic diseases		12	(2.2%)		4	(1.5%)		8	(2.9%)		0.50		54.4	(± 19.4)
VIII.	No pathognomonic histological findings^a^		23	(4.3%)		5	(1.9%)		18	(6.5%)		0.28		58.0	(± 18.3)
	Total		536	(100%)		260	(48.5%)		276	(51.5%)		0.94		50.5	(± 17.9)

Six diagnoses exceeded half (51.7%) of all histopathological diagnoses, namely: i. periapical (radicular) odontogenic cyst (24.1%, M:F = 1.8:1), ii. traumatic fibroma (11.0%), iii. mucous cyst - osteonecrosis - diagnoses with non-pathognomonic histological findings (4.3% each), and iv. odontogenic cyst of inflammatory origin with no further specification (3.7%, M:F = 1.9:1). The most common type of malignancy was oral squamous cell carcinoma (OSCC) (3.4% of all lesions, 62.1% of all malignancies including lymphomas, M:F = 1:1) (Tables 2-4).

**Table 2:** Histological diagnosis according to gender and age, grouped by diagnostic category Lesions associated with: ^a ^Squamous epithelium; ^b ^Salivary glands; ^c ^Fibrous connective tissue; ^d ^Osseous tissue; ^e ^Adipose tissue; ^f ^Muscular tissue; ^g ^Vascular tissue; ^h ^Peripheral nervous tissue; ^i ^Melanocytic origin; ^j ^Odontogenic origin; ^k ^Giant cells. Note: - denotes a missing or non-calculable value

Histological diagnosis		Gender						Age (years)			Total		% Group	% Total
		M		F		M:F		Mean	(±SD)					
I. Benign tumors and tumor-like lesions		52		92		0.57		51.0	(± 19)		144		100.0	26.9
Oral squamous papilloma^a^		3		4		0.75		55.7	(± 16.8)		7		4.9	1.3
Pleomorphic adenoma^b^		2		1		2.00		42.7	(± 11.6)		3		2.1	0.6
Monomorphous tubular adenoma^b^		0		1		0.00		78.0	-		1		0.7	0.2
Sialadenoma papilliferum^b^		0		1		0.00		35.0	-		1		0.7	0.2
Cemento-ossifying fibroma^c^		0		1		0.00		30.0	-		1		0.7	0.2
Giant cell fibroma^c^		2		1		2.00		42.3	(± 10.1)		3		2.1	0.6
Fibroepithelial tumor^c^		0		3		0.00		42.7	(± 7.2)		3		2.1	0.6
Fibroepithelial polyp^c^		1		1		1.00		47.5	(± 24.7)		2		1.4	0.4
Central ossifying fibroma^c^		0		2		0.00		32.5	(± 0.7)		2		1.4	0.4
Fibrous epoulis^c^		5		4		1.25		45.1	(± 23.3)		9		6.3	1.7
Peripheral ossifying fibroma (POF)^c^		1		1		1.00		46.5	(± 46)		2		1.4	0.4
Denture-induced fibrous hyperplasia^c^		0		2		0.00		75.0	(± 5.7)		2		1.4	0.4
Traumatic fibroma / Irritation fibroma^c^		19		40		0.48		54.3	(± 16.2)		59		41.0	11.0
Osteoma^d^		0		6		0.00		54.2	(± 23.1)		6		4.2	1.1
Fibrolipoma^e^		2		1		2.00		60.3	(± 19.4)		3		2.1	0.6
Lipoma^e^		0		1		0.00		77.0	-		1		0.7	0.2
Vascular leiomyoma^f^		1		0		-		63.0	-		1		0.7	0.2
Hemangioma^g^		4		1		4.00		54.8	(± 16.3)		5		3.5	0.9
Pyogenic granuloma^g^		1		6		0.17		42.0	(± 26)		7		4.9	1.3
Post extraction granuloma^g^		2		2		1.00		46.8	(± 24.8)		4		2.8	0.7
Lymphangioma^g^		0		2		0.00		23.5	(± 0.7)		2		1.4	0.4
Venous lake^g^		2		1		2.00		59.7	(± 15.1)		3		2.1	0.6
Granular cell tumor^h^		0		1		0.00		-	-		1		0.7	0.2
Melanocytic nevus^i^		0		1		0.00		17.0	-		1		0.7	0.2
Oral melanotic macule^i^		0		1		0.00		40.0	-		1		0.7	0.2
Ameloblastoma^j^		4		0		-		49.5	(± 20)		4		2.8	0.7
Ameloblastic fibroma^j^		0		1		0.00		17.0	-		1		0.7	0.2
Odontoma^j^		0		2		0.00		52.0	(± 35.4)		2		1.4	0.4
Central giant cell granuloma^k^		1		1		1.00		30.5	(± 6.4)		2		1.4	0.4
Peripheral giant cell granuloma^k^		2		3		0.67		61.0	(± 12.5)		5		3.5	0.9

**Table 3:** Histological diagnosis according to gender and age, grouped by diagnostic categories (cont) Tumor origin: ^a ^Squamous epithelium; ^b ^Skin; ^c ^Glandular epithelium; ^d ^Osseous tissue; ^e ^Muscular tissue; ^f ^Lung tissue. Origin of cystic lesions: ^g ^Oral soft tissue cyst; ^h ^Developmental odontogenic cysts of the jaws; ^i ^Inflammatory odontogenic cysts of the jaws; ^j ^Non-odontogenic cysts of the jaws. Note: - denotes a missing or non-calculable value.

Histological diagnosis		Gender						Age (years)			Total		% Group	% Total
		M		F		M:F		Mean	(±SD)					
II. Malignant tumors		14		14		1.00		67.9	(± 11.9)		28		100.0	5.2
Carcinoma in situ (CIS)^a^		1		0		-		65.0	-		1		3.6	0.2
Oral squamous cell carcinoma (OSCC)^a^		9		9		1.00		69.4	(± 12.9)		18		64.3	3.4
Primary intraosseous squamous cell carcinoma (PIOSCC)^a^		1		0		-		61.0	-		1		3.6	0.2
Oral carcinoma cuniculatum^a^		2		0		-		59.5	(± 6.4)		2		7.1	0.4
Basal cell carcinoma (BCC)^b^		0		1		0.00		66.0	-		1		3.6	0.2
Acinic cell carcinoma^c^		0		1		0.00		69.0	-		1		3.6	0.2
Clear cell myoepithelial carcinoma^c^		0		1		0.00		79.0	-		1		3.6	0.2
Aggressive osteoblastoma (AO) / Parosteal osteosarcoma^d^		0		1		0.00		46.0	-		1		3.6	0.2
Embryonal Rhabdomyosarcoma (ERMS)^e^		0		1		0.00		77.0	-		1		3.6	0.2
Undifferentiated carcinoma^f^		1		0		-		69.0	-		1		3.6	0.2
IΙI. PMLs – Pre-cancerous lesions		9		18		0.50		55.1	(± 12.8)		27		100.0	5.0
Epithelial dysplasia		7		12		0.58		57.6	(± 13.8)		19		70.4	3.5
Epithelial hyperplasia with a clinical diagnosis of oral leukoplakia (OL)		2		3		0.67		52.2	(± 8)		5		18.5	0.9
Pseudoepitheliomatous hyperplasia (PEM) with a clinical diagnosis of OL		0		3		0.00		43.7	(± 0.6)		3		11.1	0.6
IV. Cysts of the jaw and oral soft tissues		147		76		1.93		44.0	(± 16)		223		100.0	41.6
Ranula^g^		0		3		0.00		26.0	(± 6.9)		3		1.3	0.6
Mucocele (Mucus cyst)^g^		19		4		4.75		28.0	(± 13.6)		23		10.3	4.3
Epidermoid cyst^g^		3		0		-		45.0	(± 4.4)		3		1.3	0.6
Glandular odontogenic cyst (GOC)^h^		4		1		4.00		49.8	(± 10.8)		5		2.2	0.9
Odontogenic keratocyst (OKC)^h^		6		4		1.50		50.1	(± 19.1)		10		4.5	1.9
Dentigerous cyst^h^		7		5		1.40		39.8	(± 20.7)		12		5.4	2.2
Odontogenic cyst of inflammatory origin with no further specification^i^		13		7		1.86		47.3	(± 15.4)		20		9.0	3.7
Periapical (radicular) cyst^i^		83		46		1.80		45.2	(± 13.9)		129		57.8	24.1
Residual cyst^i^		8		3		2.67		60.2	(± 11.7)		11		4.9	2.1
Nasopalatine duct cyst / Incisive canal cyst^j^		4		3		1.33		44.6	(± 20.3)		7		3.1	1.3

**Table 4:** Histological diagnosis according to gender and age, grouped by diagnostic categories (cont) ^a ^Inflammatory disease due to mechanical or other causes; ^b ^Inflammatory diseases of the jaws; ^c ^Inflammatory diseases of salivary glands; ^d ^Inflammatory diseases of paranasal sinuses; ^e ^Pathology induced by the immune system; ^f ^Lymphoid tissue/reticuloendothelial system-associated pathology; ^g ^Metabolic disorder; ^h ^Infectious disease. Note: - denotes a missing or non calculable value.

Histological diagnosis			Gender									Age (years)			Total		% Group	% Total
			M				F			M:F		Mean	(±SD)					
V. Inflammatory diseases			24				28			0.86		52.7	(± 15.4)		52		100.0	9.7
Foreign body reaction^a^			0				2			0.00		64.5	(± 24.7)		2		3.8	0.4
Oral soft tissue abscess^a^			1				0			-		67.0	-		1		1.9	0.2
Blood clot^a^			0				2			0.00		61.5	(± 2.1)		2		3.8	0.4
Non-specific granulomatous inflammation^a^			4				5			0.80		55.9	(± 11.7)		9		17.3	1.7
Non-specific ulcer^a^			1				0			-		70.0	-		1		1.9	0.2
Subacute inflammation^a^			2				1			2.00		51.3	(± 26.4)		3		5.8	0.6
Chronic inflammation^a^			2				1			2.00		61.0	(± 17.7)		3		5.8	0.6
Periapical granuloma^b^			9				5			1.80		43.9	(± 16.1)		14		26.9	2.6
Acute osteomyelitis^b^			1				0			-		57.0	-		1		1.9	0.2
Chronic osteomyelitis^b^			0				2			0.00		47.0	(± 9.9)		2		3.8	0.4
Sclerosing osteomyelitis^b^			0				2			0.00		45.0	(± 25.5)		2		3.8	0.4
Sialadenitis^c^			0				4			0.00		52.5	(± 16.9)		4		7.7	0.7
Sialolithiasis^c^			1				0			-		66.0	-		1		1.9	0.2
Paranasal sinus mucocele^d^			0				1			0.00		67.0	-		1		1.9	0.2
Sinonasal inflammatory polyp^d^			3				2			1.50		50.4	(± 11.1)		5		9.6	0.9
Subacute sinusitis^d^			0				1			0.00		53.0	-		1		1.9	0.2
VI. Pathology induced by drugs and other factors			5				22			0.23		66.2	(± 13.9)		27		100.0	5.0
Radiation-induced oral mucositis (RIOM)			0				1			0.00		62.0	-		1		3.7	0.2
Plasma cell granuloma (PCG)			1				0			-		79.0	-		1		3.7	0.2
Osteoradionecrosis (ORN)			1				0			-		50.0	-		1		3.7	0.2
Medication-related osteonecrosis of the jaws (MRONJ)		3				20			0.15			68.8	(± 9.4)		23		85.2	4.3
Amalgam tattoo			0				1			0.00		17.0	-		1		3.7	0.2
VII. Oral manifestations of systemic diseases			4				8			0.50		54.4	(± 19.4)		12		100.0	2.2
Oral lichen planus (OLP)^e^			1				5			0.20		67.2	(± 7)		6		50.0	1.1
Precursor T-cell lymphoblastic lymphoma^f^			1				0			-		20.0	-		1		8.3	0.2
Reactive lymphoid tissue^f^			0				1			0.00		53.0	-		1		8.3	0.2
Langerhans cell histiocytosis (LCH)^f^			1				1			1.00		33.0	(± 18.4)		2		16.7	0.4
Amyloidosis^g^			0				1			0.00		71.0	-		1		8.3	0.2
Actinomycosis^h^			1				0			-		40.0	-		1		8.3	0.2

**Figure 1:**
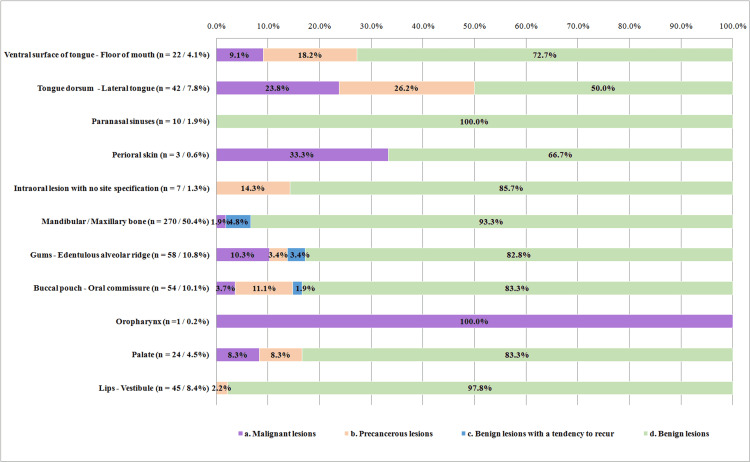
Frequency distribution of lesions by anatomic site, according to their biological behavior (Values on the Y-axis correspond to the number of lesions in each anatomic site and their respective percentage in relation to the whole sample of 536 lesions)

Depending on biological behavior, lesion frequency was distributed as follows: a. n = 29 (5.4%) for malignant lesions, b. n = 27 (5.0%) for pre-cancerous lesions, c. n = 16 (3.0%) for benign lesions with a tendency to recur, and d. n = 464 (86.6%) for benign lesions. The frequency distribution of lesions at different anatomic sites was significantly different according to their biological behavior (P<0.001). Thirty-four point five percent (34.5%) of malignant and 40.7% of pre-cancerous lesions were recorded on the tongue while 54.3% of benign lesions and 81.3% of benign lesions with a tendency to recur were recorded in the maxillary and mandibular bones (Figure 1).

Concordance between clinical and histological diagnosis was 74.9%, while concordance between clinical suspicion of pre-cancerous or malignant lesions and histological diagnosis was 96.4% for biopsies with recorded clinical diagnosis (n = 520) (Cohen's κ = 0.817, P<0.001). Sensitivity, specificity, and the positive and negative prognostic values concerning the clinical diagnosis of pre-cancerous and malignant lesions vs benign lesions were 85%, 97.7%, 81.7%, and 98.3%, respectively. In five cases of malignant lesions, no clinical suspicion of malignancy was raised.

## Discussion

The number of biopsies for the period of 8.5 years is comparable to similar studies from single surgical units in Europe and the Middle East [2,4,7] and is low in comparison to studies originating from oral and maxillofacial pathology services in Europe and USA [5,12-14]. However, the male to female ratio (0.94:1) of the present study is comparable to the study by Jones and Franklin (0.9:1) for 44,007 biopsies over a 30-year period in the UK [12].

Due to the large heterogeneity in the way samples are collected and variations in the age groups of patients and design, the recorded frequency of histological diagnoses of lesions differ between similar studies and thus direct comparisons may not be possible [4].

The significantly higher mean age of women in the study sample may explain the higher incidence of squamous cell carcinoma (M:F ratio = 1:1), as well as pre-cancerous lesions (1:2) and lichen planus (1:5) in women, compared to large studies from the UK and USA [12-13]. It has been shown that the average age of onset of OSCC in women is higher than that of men and that OSCC is more prevalent in women after the age of 70 [16]. Almoznino et al. also found an increased risk for lichen planus and epithelial dysplasia with the progression of age (OR: 1.04 and OR: 1.08, respectively, for each year of aging) [2].

A periapical (radicular) odontogenic cyst was the prevalent histological diagnosis in this study (24.1%, M:F ratio = 1.8:1). This high rate of periapical cysts in comparison to similar studies from Europe [4,12,14] may be attributed to the high number of referrals from general dentists for apicoectomies. In addition, the higher incidence of peri-apical cysts that were recorded in men is confirmed by other researchers in the Mediterranean and Middle Eastern populations [7,17]. This finding may reflect the fact that men have poorer oral hygiene than women and that they are more likely to undergo injuries in the anterior mandibular region at a young age [17]. In general, inflammatory odontogenic cysts of the jaws occurred in 104 men (40% of male lesions) and only in 56 females (20.3% of female lesions), accounting for a male to female ratio of 1.97:1 and for 29.9% of all lesions. These lesions were also prevalent at a rate of 36.3% in the work of Sklavounou et al. in a Greek population of children and adolescents [6].

The concordance rate of 74.9% between clinical and histological (definitive) diagnoses of lesions is comparable to rates recorded in the literature. Concordance between clinical and histological diagnosis following biopsy has been documented in a range from 44.6% to 84.5% and higher percentages are associated with biopsies performed by oral and maxillofacial surgeons [18-22]. In the literature, sensitivity in discerning malignant and pre-cancerous lesions vs benign lesions ranges from 93.0% to 98.1% while specificity has been recorded at a range from 31.0% to 48.6% [22-23]. The sensitivity of the clinical examination in diagnosing pre-cancerous or malignant lesions in this study is comparable to a meta-analysis of similar types of studies (85% vs 93%) while specificity is remarkably higher (97.7% vs 48.6%) [23]. Out of the 19 (3.6%) lesions for which clinical and histological diagnosis differed in such terms, five appeared to be malignant, namely, i. PIOSCC (in a 61-year-old male that resembled a large periapical cyst of the maxilla), ii. clear cell myoepithelial carcinoma (in a 79-year-old woman that resembled a residual cyst palpable to the palate), iii. acinic cell carcinoma (in a 69-year-old female that resembled benign tumors of the soft palate), iv. undifferentiated carcinoma - metastatic lung carcinoma (in a 69-year-old male with no medical record of cancer) and v. aggressive osteoblastoma/ parosteal osteosarcoma (in a 49-year-old female that resembled osteoma of the maxilla).

Concerning the histo-molecular profile of the previously described malignant neoplasms, recently published studies explored the impact of gross chromosome and specific gene numerical aberrations on their clinic-pathological features [24-26]. Additionally, epigenetic and micro-molecular factors, including hyper-hypo methylation and microRNAs, seem to be very promising markers for prognosis and targeted therapeutic strategies in subsets of patients suffering from oral malignancies [27-30].

The main limitation of the current study is the fact that the majority of cases were referrals from general dentists and other dental specialists (oral pathologists and endodontists). This issue was the determining factor for the histological types of lesions and demographic characteristics of patients in the study sample. Therefore, lesions like cysts may be overrepresented in the final sample. However, only a few studies have been published discussing oral lesion pathology incidence in Mediterranean countries [4,6-7,17] so far, and more similar studies are essential.

## Conclusions

In conclusion, our differential retrospective analysis showed that the male to female ratio was 1:1 for OSCC, 1:2 for pre-cancerous lesions, and 1:5 for lichen planus. The fact that certain lesions raised no clinical suspicion of malignancy reflects (a) the importance of histological confirmation of clinical diagnosis, (b) the importance of training in the principles and techniques of biopsy taking, and (c) the need for early referral of patients to a specialized oral and maxillofacial surgeon when malignancy is suspected.
